# The genome sequence of the Dark Umber moth, *Philereme transversata *(Hufnagel, 1767)

**DOI:** 10.12688/wellcomeopenres.24265.1

**Published:** 2025-06-02

**Authors:** Douglas Boyes, Clare Boyes, University of Oxford and Wytham Woods Genome Acquisition Lab, Darwin Tree of Life Barcoding collective, Wellcome Sanger Institute Tree of Life Management, Samples and Laboratory team, Wellcome Sanger Institute Scientific Operations: Sequencing Operations, Wellcome Sanger Institute Tree of Life Core Informatics team, Tree of Life Core Informatics collective, Darwin Tree of Life Consortium

**Affiliations:** 1 UK Centre for Ecology & Hydrology, Wallingford, England, UK; 2 Independent researcher, Welshpool, Wales, UK

**Keywords:** Philereme transversata, Dark Umber, genome sequence, chromosomal, Lepidoptera

## Abstract

We present a genome assembly from a male specimen of *Philereme transversata* (Dark Umber; Arthropoda; Insecta; Lepidoptera; Geometridae). The genome sequence has a total length of 591.75 megabases. Most of the assembly (99.1%) is scaffolded into 20 chromosomal pseudomolecules, including the Z sex chromosome. The mitochondrial genome has also been assembled, with a length of 16.07 kilobases. Gene annotation of this assembly on Ensembl identified 12,207 protein-coding genes.

## Species taxonomy

Eukaryota; Opisthokonta; Metazoa; Eumetazoa; Bilateria; Protostomia; Ecdysozoa; Panarthropoda; Arthropoda; Mandibulata; Pancrustacea; Hexapoda; Insecta; Dicondylia; Pterygota; Neoptera; Endopterygota; Amphiesmenoptera; Lepidoptera; Glossata; Neolepidoptera; Heteroneura; Ditrysia; Obtectomera; Geometroidea; Geometridae; Larentiinae; *Philereme*; *Philereme transversata* (Hufnagel, 1767) (NCBI:txid873512)

## Background

The Dark Umber ( *Philereme transversata*) is in the family Geometridae. It is a moth of woodlands and scrub, and is frequent across southern England, although rare in Devon and Cornwall. It becomes scarcer further north. It has declined in abundance since 1970 ( Randle *et al.*, 2019). It is present throughout Europe ( GBIF Secretariat, 2023).

The moth is a rich brown with a darker central cross band. The trailing edges of the wings are distinctively scalloped. There is one generation a year which flies between July and August. The caterpillars feed at night on Buckthorn and probably Alder Buckthorn, and then pupate in a cocoon in the ground ( Waring *et al.*, 2017).

**Figure 1.**
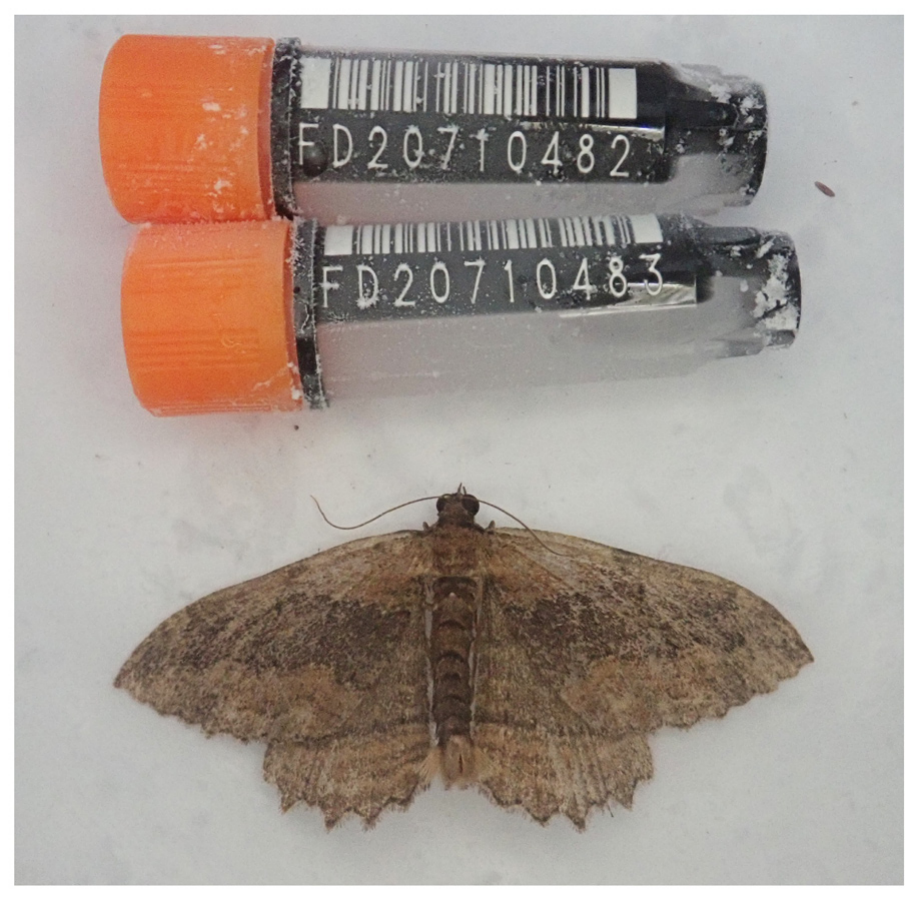
Photograph of the *Philereme transversata* (ilPhiTran2) specimen used for genome sequencing.

As part of the Darwin Tree of Life Project – which aims to generate high-quality reference genomes for all named eukaryotic species in Britain and Ireland to .to support research, conservation, and the sustainable management of biodiversity – we present a chromosomally complete genome sequence for the Dark Umber, *Philereme transversata*. This genome was assembled using the Tree of Life pipeline from a specimen collected in Wytham Woods, Oxfordshire, United Kingdom ( Figure 1).

## Genome sequence report

### Sequencing data

The genome of a specimen of *Philereme transversata* ( Figure 1) was sequenced using Pacific Biosciences single-molecule HiFi long reads, generating 61.42 Gb (gigabases) from 6.12 million reads, which were used to assemble the genome. GenomeScope analysis estimated the haploid genome size at 583.27 Mb, with a heterozygosity of 0.67% and repeat content of 35.69%. These estimates guided expectations for the assembly. Based on the estimated genome size, the sequencing data provided approximately 103 coverage. Hi-C sequencing produced 109.02 Gb from 722.01 million reads, used to scaffold the assembly. Table 1 summarises the specimen and sequencing details.

**Table 1.** Specimen and sequencing data for *Philereme transversata*.

Project information			
**Study title**	Philereme transversata (dark umber)		
**Umbrella BioProject**	PRJEB65731		
**Species**	*Philereme transversata*		
**BioSpecimen**	SAMEA10978949		
**NCBI taxonomy ID**	873512		
Specimen information			
**Technology**	**ToLID**	**BioSample accession**	**Organism part**
**PacBio long read sequencing**	ilPhiTran2	SAMEA10979299	head and thorax
**Hi-C sequencing**	ilPhiTran1	SAMEA7701748	head and thorax
Sequencing information			
**Platform**	**Run accession**	**Read count**	**Base count (Gb)**
**Hi-C Illumina NovaSeq 6000**	ERR12035307	7.22e+08	109.02
**PacBio Revio**	ERR12015773	6.12e+06	61.42

### Assembly statistics

The primary haplotype was assembled, and contigs corresponding to an alternate haplotype were also deposited in INSDC databases. The assembly was improved by manual curation, which corrected three misjoins or missing joins and removed three haplotypic duplications. These interventions reduced the total assembly length by 0.83% and decreased the scaffold count by 3.33%. The final assembly has a total length of 591.75 Mb in 57 scaffolds, with 68 gaps, and a scaffold N50 of 29.48 Mb ( Table 2).

**Table 2.** Genome assembly data for *Philereme transversata*.

Genome assembly		
Assembly name	ilPhiTran2.1	
Assembly accession	GCA_963924445.1	
*Alternate haplotype accession*	*GCA_963924475.1*	
Assembly level for primary assembly	chromosome	
Span (Mb)	591.75	
Number of contigs	125	
Number of scaffolds	57	
Longest scaffold (Mb)	41.65	
Assembly metric	Measure	*Benchmark*
Contig N50 length	9.52 Mb	≥ *1 Mb*
Scaffold N50 length	29.48 Mb	= *chromosome N50*
Consensus quality (QV)	Primary: 65.4; alternate: 64.8; combined: 65.0	≥ *40*
*k*-mer completeness	Primary: 84.47%; alternate: 84.18%; combined: 99.64%	≥ *95%*
BUSCO *	C:98.4%[S:97.8%,D:0.6%], F:0.3%,M:1.3%,n:5,286	*S > 90%; D < 5%*
Percentage of assembly assigned to chromosomes	99.1%	≥ *90%*
Sex chromosomes	Z	*localised homologous pairs*
Organelles	Mitochondrial genome: 16.07 kb	*complete single alleles*

* BUSCO scores based on the lepidoptera_odb10 BUSCO set using version 5.5.0. C = complete [S = single copy, D = duplicated], F = fragmented, M = missing, n = number of orthologues in comparison.

**Figure 2.**
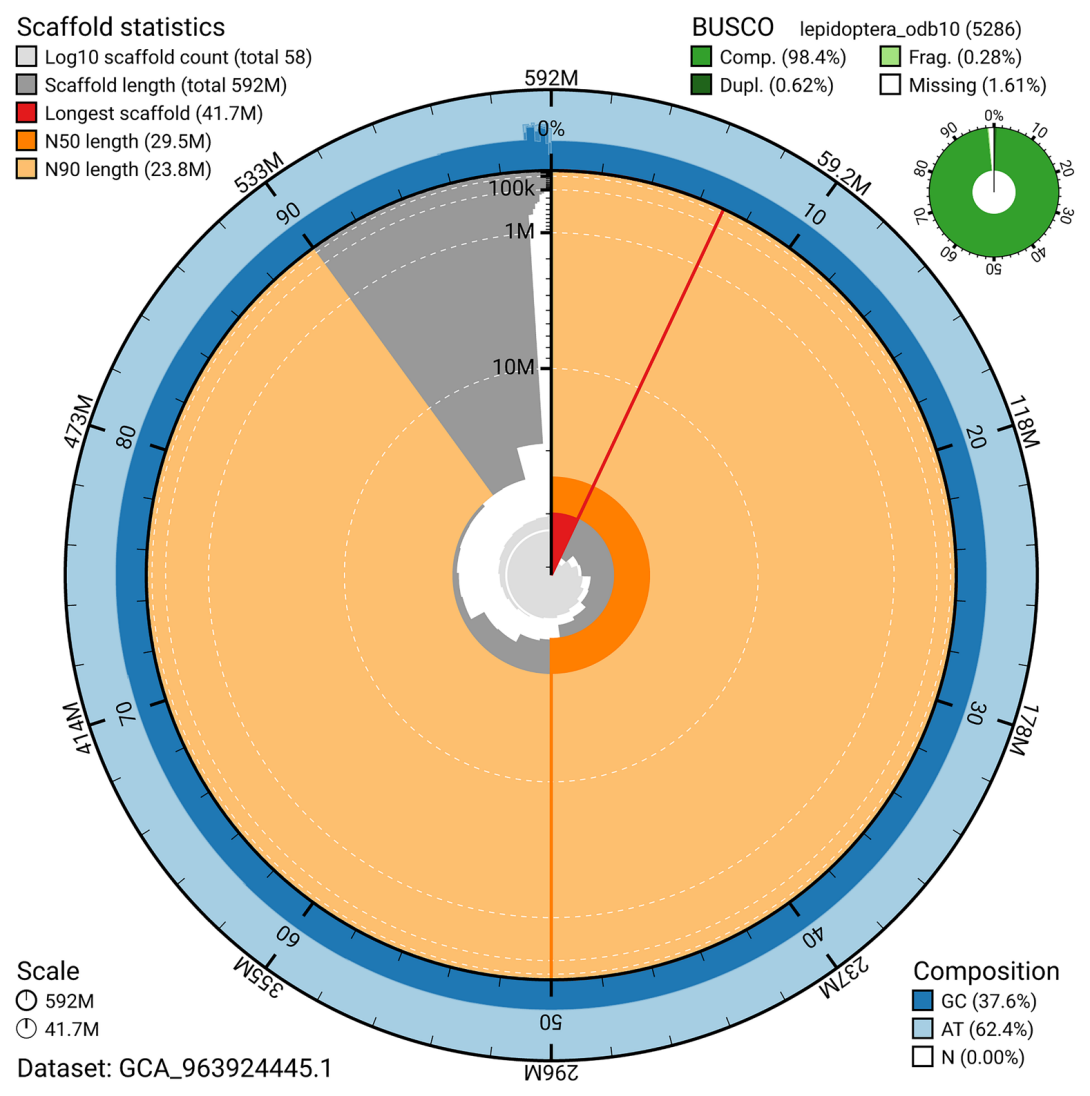
Genome assembly of *Philereme transversata*, ilPhiTran2.1: metrics. The BlobToolKit snail plot provides an overview of assembly metrics and BUSCO gene completeness. The circumference represents the length of the whole genome sequence, and the main plot is divided into 1,000 bins around the circumference. The outermost blue tracks display the distribution of GC, AT, and N percentages across the bins. Scaffolds are arranged clockwise from longest to shortest and are depicted in dark grey. The longest scaffold is indicated by the red arc, and the deeper orange and pale orange arcs represent the N50 and N90 lengths. A light grey spiral at the centre shows the cumulative scaffold count on a logarithmic scale. A summary of complete, fragmented, duplicated, and missing BUSCO genes in the lepidoptera_odb10 set is presented at the top right. An interactive version of this figure is available at https://blobtoolkit.genomehubs.org/view/GCA_963924445.1/dataset/GCA_963924445.1/snail.

**Figure 3.**
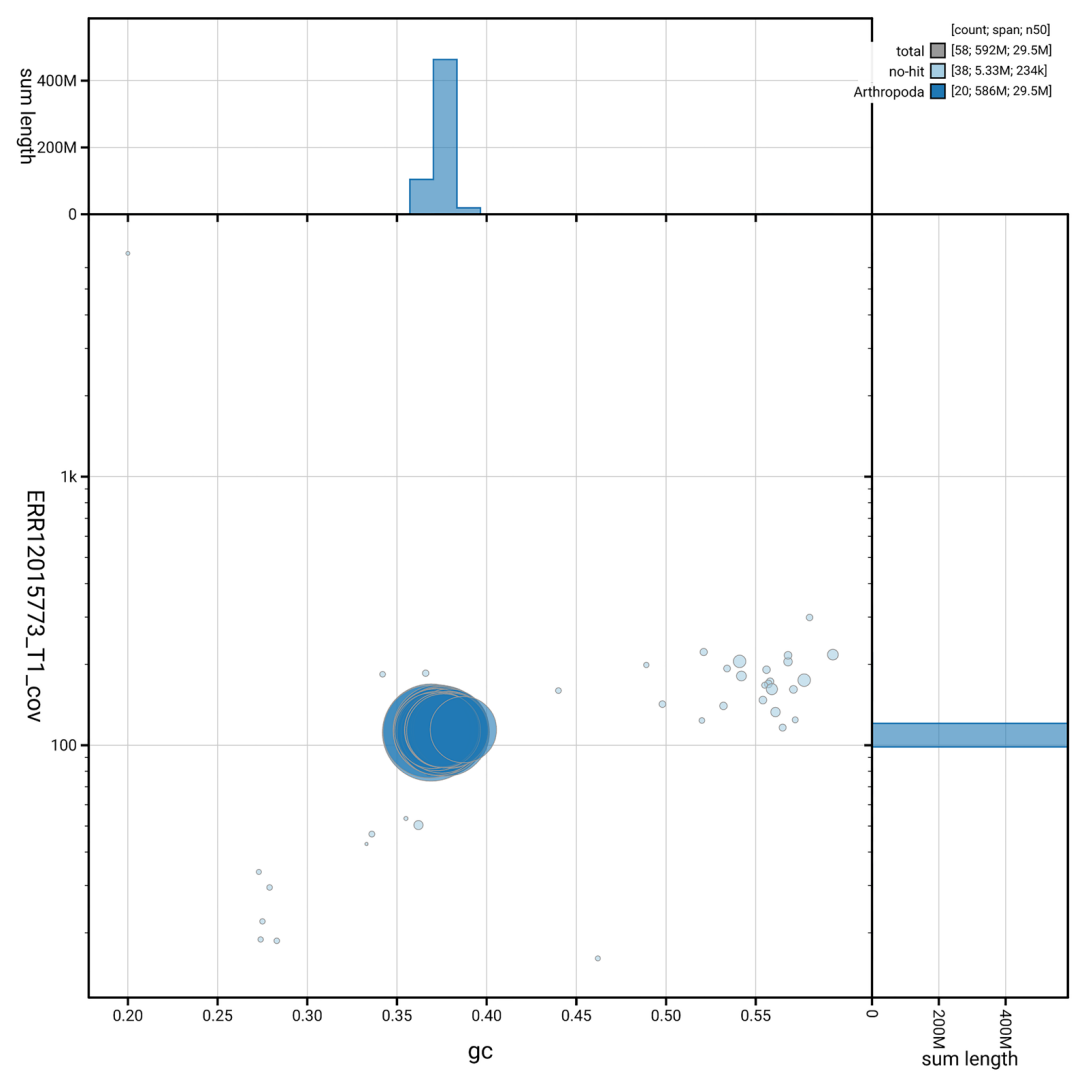
Genome assembly of *Philereme transversata*, ilPhiTran2.1: BlobToolKit GC-coverage plot. Blob plot showing sequence coverage (vertical axis) and GC content (horizontal axis). The circles represent scaffolds, with the size proportional to scaffold length and the colour representing phylum membership. The histograms along the axes display the total length of sequences distributed across different levels of coverage and GC content. An interactive version of this figure is available at https://blobtoolkit.genomehubs.org/view/GCA_963924445.1/dataset/GCA_963924445.1/blob.

**Figure 4.**
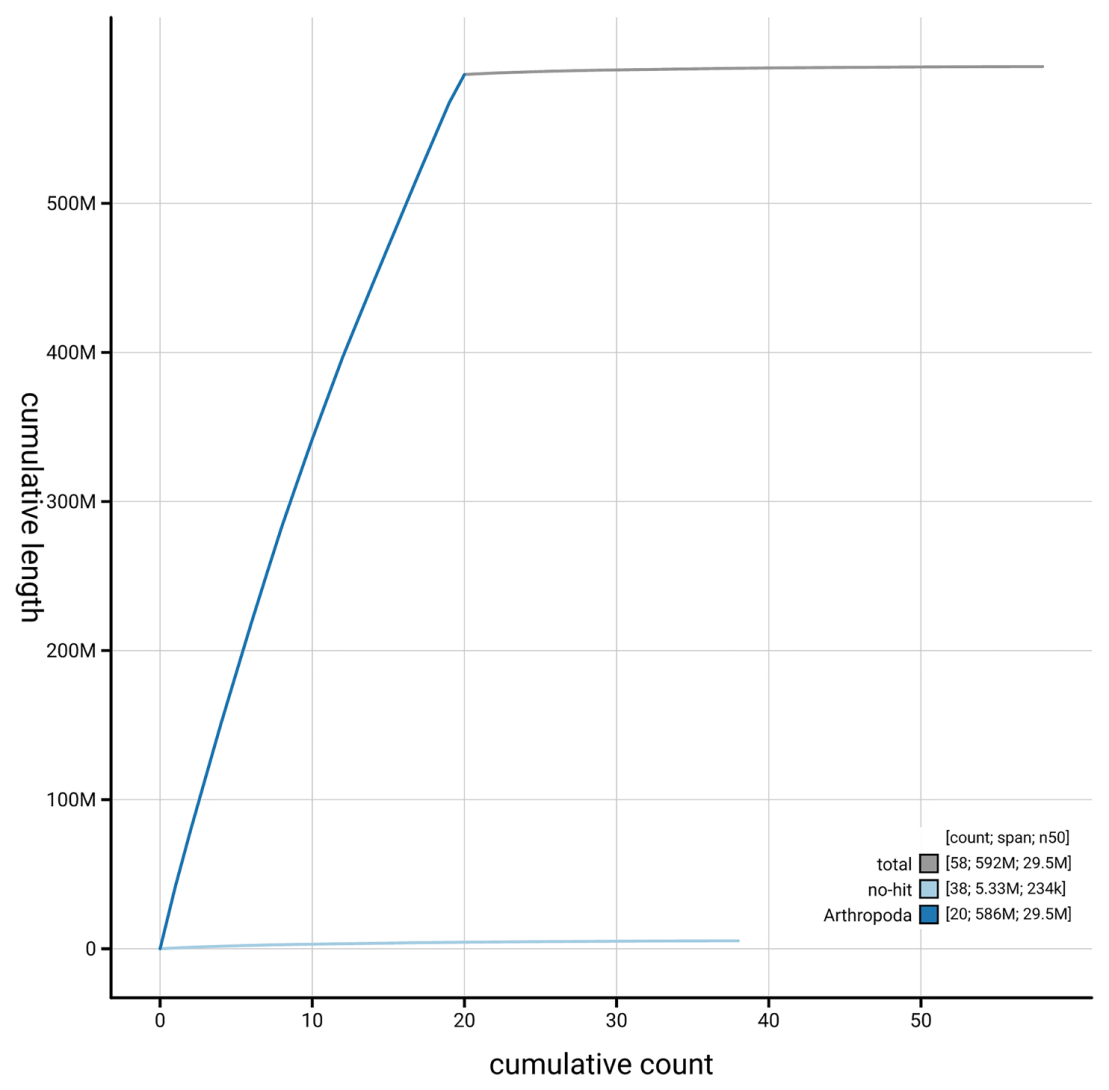
Genome assembly of *Philereme transversata,* ilPhiTran2.1: BlobToolKit cumulative sequence plot. The grey line shows cumulative length for all scaffolds. Coloured lines show cumulative lengths of scaffolds assigned to each phylum using the buscogenes taxrule. An interactive version of this figure is available at https://blobtoolkit.genomehubs.org/view/GCA_963924445.1/dataset/GCA_963924445.1/cumulative.

The snail plot in Figure 2 provides a summary of the assembly statistics, indicating the distribution of scaffold lengths and other assembly metrics. Figure 3 shows the distribution of scaffolds by GC proportion and coverage. Figure 4 presents a cumulative assembly plot, with separate curves representing different scaffold subsets assigned to various phyla, illustrating the completeness of the assembly.

**Figure 5.**
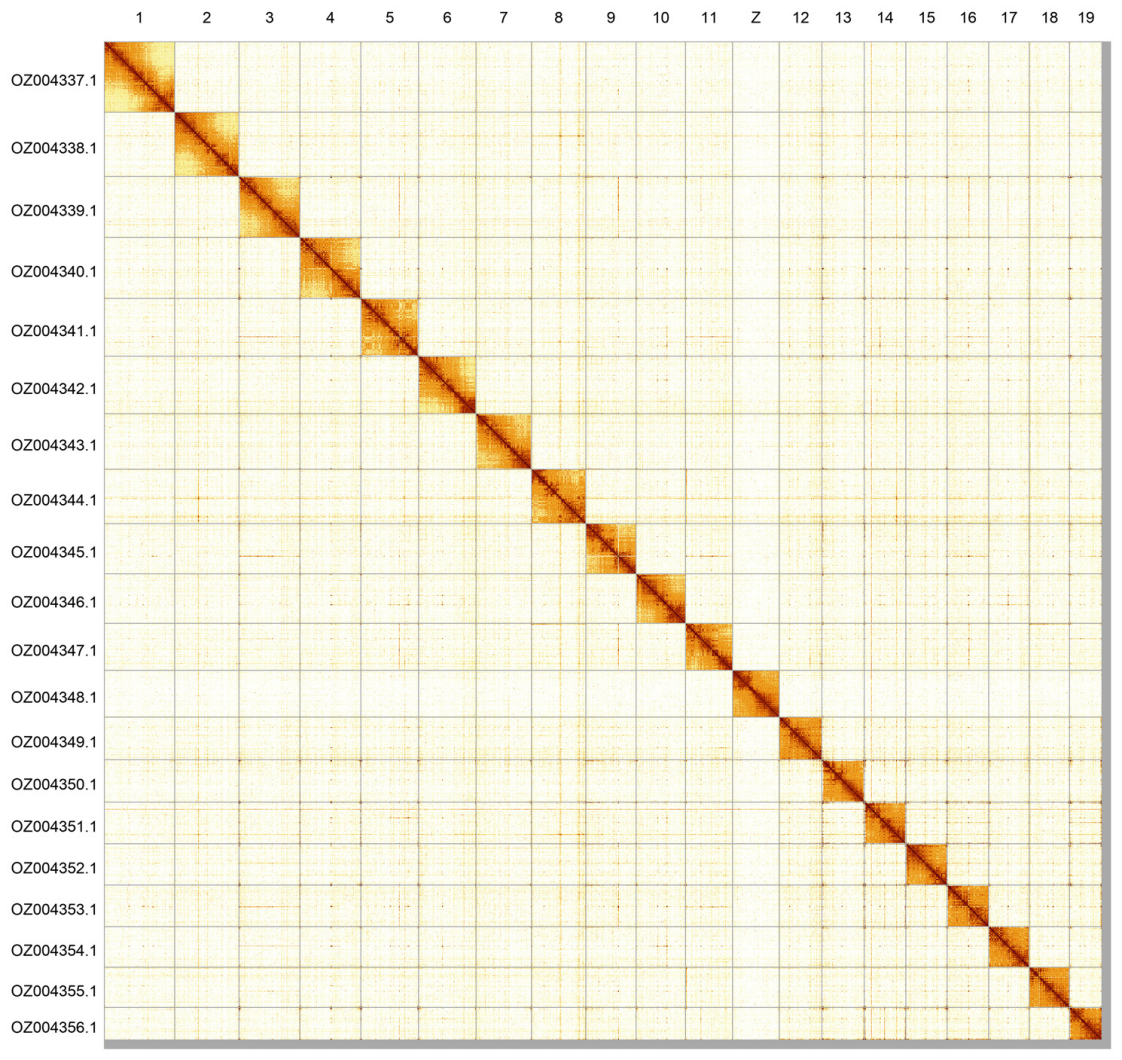
Genome assembly of *Philereme transversata*: Hi-C contact map of the ilPhiTran2.1 assembly, generated using PretextSnapshot. Chromosomes are shown in order of size and labelled with chromosome numbers (top) and chromosome accession numbers (left).

Most of the assembly sequence (99.1%) was assigned to 20 chromosomal-level scaffolds, representing 19 autosomes and the Z sex chromosome. These chromosome-level scaffolds, confirmed by Hi-C data, are named according to size ( Figure 5; Table 3). During curation, the Z chromosome was assigned based on synteny to *Philereme vetulata* (GCA_918857605.2).

**Table 3.** Chromosomal pseudomolecules in the genome assembly of *Philereme transversata*, ilPhiTran2.

INSDC accession	Name	Length (Mb)	GC%
OZ004337.1	1	41.65	37
OZ004338.1	2	37.73	37
OZ004339.1	3	35.9	37.5
OZ004340.1	4	35.67	37.5
OZ004341.1	5	33.95	37.5
OZ004342.1	6	33.78	37.5
OZ004343.1	7	32.56	37
OZ004344.1	8	31.97	37
OZ004345.1	9	29.48	37.5
OZ004346.1	10	29.14	37.5
OZ004347.1	11	27.59	38
OZ004349.1	12	25.0	37
OZ004350.1	13	24.82	38
OZ004351.1	14	24.5	38
OZ004352.1	15	24.37	38
OZ004353.1	16	24.32	37.5
OZ004354.1	17	23.83	37.5
OZ004355.1	18	23.77	37.5
OZ004356.1	19	18.97	38.5
OZ004348.1	Z	27.43	37
OZ004357.1	MT	0.02	21

The mitochondrial genome was also assembled. This sequence is included as a contig in the multifasta file of the genome submission and as a standalone record.

### Assembly quality metrics

The estimated Quality Value (QV) and *k*-mer completeness metrics, along with BUSCO completeness scores, were calculated for each haplotype and the combined assembly. The QV reflects the base-level accuracy of the assembly, while *k*-mer completeness indicates the proportion of expected *k*-mers identified in the assembly. BUSCO scores provide a measure of completeness based on benchmarking universal single-copy orthologues.

The combined primary and alternate assemblies achieve an estimated QV of 65.0. The *k*-mer completeness is 84.47% for the primary haplotype and 84.18% for the alternate haplotype; and 99.64% for the combined primary and alternate assemblies. BUSCO v.5.5.0 analysis using the lepidoptera_odb10 reference set ( *n* = 5,286) identified 98.4% of the expected gene set (single = 97.8%, duplicated = 0.6%).

Table 2 provides assembly metric benchmarks adapted from Rhie *et al.* (2021) and the Earth BioGenome Project Report on Assembly Standards September 2024. The primary assembly achieves the EBP reference standard of **6.C.Q65**.

## Genome annotation report

The *Philereme transversata* genome assembly (GCA_963924445.1) was annotated externally by Ensembl at the European Bioinformatics Institute (EBI). This annotation includes 21,451 transcribed mRNAs from 12,207 protein-coding and 1,934 non-coding genes. The average transcript length is 18,457.27 bp. There are 1.52 coding transcripts per gene and 7.48 exons per transcript. For further information about the annotation, please refer to https://beta.ensembl.org/species/5ce8e749-aef4-4d48-93df-a5d729e2e8cc.

## Methods

### Sample acquisition and DNA barcoding

The specimen used for genome sequencing was an adult male *Philereme transversata* (specimen ID Ox001682, ToLID ilPhiTran2), collected from Wytham Woods, Oxfordshire, United Kingdom (latitude 51.772, longitude -1.338) on 2021-07-17, using a light trap. A second specimen was used for Hi-C sequencing (specimen ID Ox000695, ToLID ilPhiTran1). This specimen was collected from the same location on 2020-07-20, using a light trap. Both specimens were collected and identified by Douglas Boyes and preserved on dry ice.

The initial identification was verified by an additional DNA barcoding process according to the framework developed by Twyford *et al.* (2024). A small sample was dissected from each specimen and stored in ethanol, while the remaining parts were shipped on dry ice to the Wellcome Sanger Institute (WSI) ( Pereira *et al.*, 2022). The tissue was lysed, the COI marker region was amplified by PCR, and amplicons were sequenced and compared to the BOLD database, confirming the species identification ( Crowley *et al.*, 2023). Following whole genome sequence generation, the relevant DNA barcode region was also used alongside the initial barcoding data for sample tracking at the WSI ( Twyford *et al.*, 2024). The standard operating procedures for Darwin Tree of Life barcoding have been deposited on protocols.io ( Beasley *et al.*, 2023).

Metadata collection for samples adhered to the Darwin Tree of Life project standards described by Lawniczak *et al*. (2022).

### Nucleic acid extraction

The workflow for high molecular weight (HMW) DNA extraction at the Wellcome Sanger Institute (WSI) Tree of Life Core Laboratory includes a sequence of procedures: sample preparation and homogenisation, DNA extraction, fragmentation and purification ( Howard *et al.*, 2025). Detailed protocols are available on protocols.io ( Denton *et al.*, 2023b). The ilPhiTran2 sample was prepared for DNA extraction by weighing and dissecting it on dry ice ( Jay *et al.*, 2023). Tissue from the head and thorax was homogenised using a PowerMasher II tissue disruptor ( Denton *et al.*, 2023a).

HMW DNA was extracted in the WSI Scientific Operations core using the Automated MagAttract v2 protocol ( Oatley *et al.*, 2023). The DNA was sheared into an average fragment size of 12–20 kb in a Megaruptor 3 system ( Bates *et al.*, 2023). Sheared DNA was purified by solid-phase reversible immobilisation, using AMPure PB beads to eliminate shorter fragments and concentrate the DNA ( Strickland *et al.*, 2023). The concentration of the sheared and purified DNA was assessed using a Nanodrop spectrophotometer and Qubit Fluorometer using the Qubit dsDNA High Sensitivity Assay kit. Fragment size distribution was evaluated by running the sample on the FemtoPulse system.

### Hi-C sample preparation and crosslinking

Hi-C data were generated from the head and thorax of the ilPhiTran1 sample using the Arima-HiC v2 kit (Arima Genomics) with 20–50 mg of frozen tissue (stored at –80 °C). As per manufacturer’s instructions, tissue was fixed, and the DNA crosslinked using a TC buffer with 22% formaldehyde concentration, and a final formaldehyde concentration of 2%. The tissue was then homogenised using the Diagnocine Power Masher-II. The crosslinked DNA was digested using a restriction enzyme master mix, then biotinylated and ligated. A clean up was performed with SPRIselect beads prior to library preparation. DNA concentration was quantified using the Qubit Fluorometer v4.0 (Thermo Fisher Scientific) and Qubit HS Assay Kit, and sample biotinylation percentage was estimated using the Arima-HiC v2 QC beads.

### Library preparation and sequencing

Library preparation and sequencing were performed at the WSI Scientific Operations core.

***PacBio HiFi***

At a minimum, samples were required to have an average fragment size exceeding 8 kb and a total mass over 400 ng to proceed to the low-input SMRTbell Prep Kit 3.0 protocol (Pacific Biosciences), depending on genome size and sequencing depth required. Libraries were prepared using the SMRTbell Prep Kit 3.0 as per the manufacturer's instructions. The kit includes the reagents required for end repair/A-tailing, adapter ligation, post-ligation SMRTbell bead cleanup, and nuclease treatment. Size-selection and clean-up were carried out using diluted AMPure PB beads (Pacific Biosciences). DNA concentration was quantified using the Qubit Fluorometer v4.0 (ThermoFisher Scientific) with Qubit 1X dsDNA HS assay kit and the final library fragment size analysis was carried out using the Agilent Femto Pulse Automated Pulsed Field CE Instrument (Agilent Technologies) and the gDNA 55kb BAC analysis kit.

Samples were sequenced on a Revio instrument (Pacific Biosciences, California, USA). Prepared libraries were normalised to 2 nM, and 15 μL was used for making complexes. Primers were annealed and polymerases were bound to create circularised complexes according to manufacturer’s instructions. The complexes were purified with the 1.2X clean up with SMRTbell beads. The purified complexes were then diluted to the Revio loading concentration (in the range 200–300 pM), and spiked with a Revio sequencing internal control. Samples were sequenced on Revio 25M SMRT cells (Pacific Biosciences, California, USA). The SMRT link software, a PacBio web-based end-to-end workflow manager, was used to set-up and monitor the run, as well as perform primary and secondary analysis of the data upon completion.

***Hi-C***

For Hi-C library preparation, the biotinylated DNA constructs were fragmented using a Covaris E220 sonicator and size-selected to 400–600 bp using SPRISelect beads. DNA was then enriched using Arima-HiC v2 Enrichment beads. The NEBNext Ultra II DNA Library Prep Kit (New England Biolabs) was used for end repair, A-tailing, and adapter ligation, following a modified protocol in which library preparation is carried out while the DNA remains bound to the enrichment beads. PCR amplification was performed using KAPA HiFi HotStart mix and custom dual-indexed adapters (Integrated DNA Technologies) in a 96-well plate format. Depending on sample concentration and biotinylation percentage determined at the crosslinking stage, samples were amplified for 10–16 PCR cycles. Post-PCR clean-up was carried out using SPRISelect beads. The libraries were quantified using the Accuclear Ultra High Sensitivity dsDNA Standards Assay kit (Biotium) and normalised to 10 ng/μL before sequencing. Hi-C sequencing was performed on the Illumina NovaSeq 6000 instrument using 150 bp paired-end reads.

### Genome assembly, curation and evaluation

***Assembly***

Prior to assembly of the PacBio HiFi reads, a database of *k*-mer counts ( *k* = 31) was generated from the filtered reads using FastK. GenomeScope2 ( Ranallo-Benavidez *et al.*, 2020) was used to analyse the *k*-mer frequency distributions, providing estimates of genome size, heterozygosity, and repeat content.

The HiFi reads were first assembled using Hifiasm ( Cheng *et al.*, 2021) with the --primary option. Haplotypic duplications were identified and removed using purge_dups ( Guan *et al.*, 2020). The Hi-C reads ( Rao *et al.*, 2014) were mapped to the primary contigs using bwa-mem2 ( Vasimuddin *et al.*, 2019), and the contigs were scaffolded in YaHS ( Zhou *et al.*, 2023) using the --break option for handling potential misassemblies. The scaffolded assemblies were evaluated using Gfastats ( Formenti *et al.*, 2022), BUSCO ( Manni *et al.*, 2021) and MERQURY.FK ( Rhie *et al.*, 2020).

The mitochondrial genome was assembled using MitoHiFi ( Uliano-Silva *et al.*, 2023), which runs MitoFinder ( Allio *et al.*, 2020) and uses these annotations to select the final mitochondrial contig and to ensure the general quality of the sequence.

***Assembly curation***

The assembly was decontaminated using the Assembly Screen for Cobionts and Contaminants (ASCC) pipeline. Flat files and maps used in curation were generated via the TreeVal pipeline ( Pointon *et al.*, 2023). Manual curation was conducted primarily in PretextView ( Harry, 2022) and HiGlass ( Kerpedjiev *et al.*, 2018), with additional insights provided by JBrowse2 ( Diesh *et al.*, 2023). Scaffolds were visually inspected and corrected as described by Howe *et al*. (2021). Any identified contamination, missed joins, and mis-joins were amended, and duplicate sequences were tagged and removed. The curation process is documented at https://gitlab.com/wtsi-grit/rapid-curation.

***Assembly quality assessment***

The Merqury.FK tool ( Rhie *et al.*, 2020), run in a Singularity container ( Kurtzer *et al.*, 2017), was used to evaluate *k*-mer completeness and assembly quality for the primary and alternate haplotypes using the *k*-mer databases ( *k* = 31) computed prior to genome assembly. The analysis outputs included assembly QV scores and completeness statistics.

The genome was analysed in the blobtoolkit pipeline, a Nextflow ( Di Tommaso *et al.*, 2017) port of the previous Snakemake Blobtoolkit pipeline ( Challis *et al.*, 2020). It aligns the PacBio reads in SAMtools ( Danecek *et al.*, 2021) and minimap2 ( Li, 2018) and generates coverage tracks for regions of fixed size. In parallel, it queries the GoaT database ( Challis *et al.*, 2023) to identify all matching BUSCO lineages to run BUSCO ( Manni *et al.*, 2021). For the three domain-level BUSCO lineages, the pipeline aligns the BUSCO genes to the UniProt Reference Proteomes database ( Bateman *et al.*, 2023) with DIAMOND blastp ( Buchfink *et al.*, 2021). The genome is also divided into chunks according to the density of the BUSCO genes from the closest taxonomic lineage, and each chunk is aligned to the UniProt Reference Proteomes database using DIAMOND blastx. Genome sequences without a hit are chunked using seqtk and aligned to the NT database with blastn ( Altschul *et al.*, 1990). The blobtools suite combines all these outputs into a blobdir for visualisation.

The blobtoolkit pipeline was developed using nf-core tooling ( Ewels *et al.*, 2020) and MultiQC ( Ewels *et al.*, 2016), relying on the Conda package manager, the Bioconda initiative ( Grüning *et al.*, 2018), the Biocontainers infrastructure ( da Veiga Leprevost *et al.*, 2017), as well as the Docker ( Merkel, 2014) and Singularity ( Kurtzer *et al.*, 2017) containerisation solutions.

Table 4 contains a list of relevant software tool versions and sources.

**Table 4.** Software tools: versions and sources.

Software tool	Version	Source
BLAST	2.14.0	ftp://ftp.ncbi.nlm.nih.gov/blast/executables/blast+/
BlobToolKit	4.3.9	https://github.com/blobtoolkit/blobtoolkit
BUSCO	5.5.0	https://gitlab.com/ezlab/busco
bwa-mem2	2.2.1	https://github.com/bwa-mem2/bwa-mem2
DIAMOND	2.1.8	https://github.com/bbuchfink/diamond
fasta_windows	0.2.4	https://github.com/tolkit/fasta_windows
FastK	666652151335353eef2fcd58880bcef5bc2928e1	https://github.com/thegenemyers/FASTK
GenomeScope2.0	2.0.1	https://github.com/tbenavi1/genomescope2.0
Gfastats	1.3.6	https://github.com/vgl-hub/gfastats
GoaT CLI	0.2.5	https://github.com/genomehubs/goat-cli
Hifiasm	0.19.5-r587	https://github.com/chhylp123/hifiasm
HiGlass	44086069ee7d4d3f6f3f0012569789ec138f42b84aa44357826c0b6753eb28de	https://github.com/higlass/higlass
MerquryFK	d00d98157618f4e8d1a9190026b19b471055b22e	https://github.com/thegenemyers/MERQURY.FK
Minimap2	2.24-r1122	https://github.com/lh3/minimap2
MitoHiFi	3	https://github.com/marcelauliano/MitoHiFi
MultiQC	1.14, 1.17, and 1.18	https://github.com/MultiQC/MultiQC
Nextflow	23.04.1	https://github.com/nextflow-io/nextflow
PretextSnapshot	-	https://github.com/sanger-tol/PretextSnapshot
PretextView	0.2.5	https://github.com/sanger-tol/PretextView
purge_dups	1.2.5	https://github.com/dfguan/purge_dups
samtools	1.19.2	https://github.com/samtools/samtools
sanger-tol/ascc	0.1.0	https://github.com/sanger-tol/ascc
sanger-tol/blobtoolkit	0.4.0	https://github.com/sanger-tol/blobtoolkit
Seqtk	1.3	https://github.com/lh3/seqtk
Singularity	3.9.0	https://github.com/sylabs/singularity
TreeVal	1.2.0	https://github.com/sanger-tol/treeval
YaHS	1.2a.2	https://github.com/c-zhou/yahs

### Wellcome Sanger Institute – Legal and Governance

The materials that have contributed to this genome note have been supplied by a Darwin Tree of Life Partner. The submission of materials by a Darwin Tree of Life Partner is subject to the **‘Darwin Tree of Life Project Sampling Code of Practice’**, which can be found in full on the Darwin Tree of Life website here. By agreeing with and signing up to the Sampling Code of Practice, the Darwin Tree of Life Partner agrees they will meet the legal and ethical requirements and standards set out within this document in respect of all samples acquired for, and supplied to, the Darwin Tree of Life Project.

Further, the Wellcome Sanger Institute employs a process whereby due diligence is carried out proportionate to the nature of the materials themselves, and the circumstances under which they have been/are to be collected and provided for use. The purpose of this is to address and mitigate any potential legal and/or ethical implications of receipt and use of the materials as part of the research project, and to ensure that in doing so we align with best practice wherever possible. The overarching areas of consideration are:

•    Ethical review of provenance and sourcing of the material

•    Legality of collection, transfer and use (national and international)

Each transfer of samples is further undertaken according to a Research Collaboration Agreement or Material Transfer Agreement entered into by the Darwin Tree of Life Partner, Genome Research Limited (operating as the Wellcome Sanger Institute), and in some circumstances other Darwin Tree of Life collaborators.

## Data Availability

European Nucleotide Archive: Philereme transversata (dark umber). Accession number PRJEB65731; https://identifiers.org/ena.embl/PRJEB65731. The genome sequence is released openly for reuse. The *Philereme transversata* genome sequencing initiative is part of the Darwin Tree of Life Project (PRJEB40665), Sanger Institute Tree of Life Programme (PRJEB43745) and Project Psyche (PRJEB71705). All raw sequence data and the assembly have been deposited in INSDC databases. Raw data and assembly accession identifiers are reported in Table 1 and Table 2.

## Author information

Members of the University of Oxford and Wytham Woods Genome Acquisition Lab are listed here: https://doi.org/10.5281/zenodo.12157525.

Members of the Natural History Museum Genome Acquisition Lab are listed here: https://doi.org/10.5281/zenodo.12159242.

Members of the Darwin Tree of Life Barcoding collective are listed here: https://doi.org/10.5281/zenodo.12158331.

Members of the Wellcome Sanger Institute Tree of Life Management, Samples and Laboratory team are listed here: https://doi.org/10.5281/zenodo.12162482.

Members of Wellcome Sanger Institute Scientific Operations: Sequencing Operations are listed here: https://doi.org/10.5281/zenodo.14870789.

Members of the Wellcome Sanger Institute Tree of Life Core Informatics team are listed here: https://doi.org/10.5281/zenodo.12160324.

Members of the Tree of Life Core Informatics collective are listed here: https://doi.org/10.5281/zenodo.12205391.

Members of the Darwin Tree of Life Consortium are listed here: https://doi.org/10.5281/zenodo.4783558.
