# Supplementary material for: The genome sequence of the Dark Umber moth, Philereme transversata (Hufnagel, 1767)
Source: Wellcome Open Res. 2025 Jun 2;10:300. [Version 1] doi: 10.12688/wellcomeopenres.24265.1 (PMC12280868; doi:10.12688/wellcomeopenres.24265.1)
Supplement: Peer review history

# Reviewer report: Reviewer response for version 1

**DOI:** 10.21956/wellcomeopenres.26762.r124826

**Published:** 2025-07-21

**Authors:** Arun Arumugaperumal

**Affiliations:** 1 Rajalakshmi Engineering College, Chennai, Tamil Nadu, India

This is the first report of the high quality genome sequence of *Philereme transversata* also known as the Dark Umber moth. The assembly reported herewith is of size 591.75 Mb and the mitogenome is of size 16.07 kb. The authors also annotated a total of 12,207 protein coding genes. The photograph included as figure 1 is very clear and will be very helpful for readers. 

 "As part of the Darwin Tree of Life Project – which aims to generate high-quality reference genomes for all named eukaryotic species in Britain and Ireland to .to". Remove the extra 'to'.

 The link given in figure 3 & 4 and on the same page. The authors can correct the link at this stage of the publication process.

 The manual curation has brought the assembly length to 591.75 Mb (after 3.33% decrease). But what was the change in length after decontamination using ASCC pipeline?

 Did the authors use transcriptomic data to improve the assembly?

Are sufficient details of methods and materials provided to allow replication by others?

Partly

Is the rationale for creating the dataset(s) clearly described?

Yes

Are the datasets clearly presented in a useable and accessible format?

Yes

Are the protocols appropriate and is the work technically sound?

Yes

Reviewer Expertise:

Bioinformatics; Genomics

I confirm that I have read this submission and believe that I have an appropriate level of expertise to confirm that it is of an acceptable scientific standard, however I have significant reservations, as outlined above.

# Reviewer report: Reviewer response for version 1

**DOI:** 10.21956/wellcomeopenres.26762.r124818

**Published:** 2025-06-28

**Authors:** Annabel Whibley

**Affiliations:** 1 Grapevine Improvement, Bragato Research Institute, Lincoln, Lincoln, New Zealand

Boyes and colleagues report the genome assembly and genome annotation of the Dark Umber moth ( *Philereme transversata*). Overall, this is a high quality assembly (achieving EBP standard  6.C.Q65). Over 99% of the assembly has been scaffolded into 20 pseudochromosomes with excellent QV and completeness. Specimen ID has been validated by barcode sequencing.  The Data Note follows Darwin Tree of Life project protocols, assembly and annotation pipelines and reporting templates. Appropriate methods are used, metadata is comprehensive and public accession links are functional.

 There is a minor typo in the background section (insertion of “ .to”) in this sentence:   “… high-quality reference genomes for all named eukaryotic species in Britain and Ireland to .to support research…”

 I would also query the reporting of the average transcript length as >18kb. This seems unfeasibly high. Could the meaning of this statistic be clarified in the template?

Are sufficient details of methods and materials provided to allow replication by others?

Yes

Is the rationale for creating the dataset(s) clearly described?

Yes

Are the datasets clearly presented in a useable and accessible format?

Yes

Are the protocols appropriate and is the work technically sound?

Yes

Reviewer Expertise:

Genomics, Evolution, Bioinformatics

I confirm that I have read this submission and believe that I have an appropriate level of expertise to confirm that it is of an acceptable scientific standard.

# Reviewer report: Reviewer response for version 1

**DOI:** 10.21956/wellcomeopenres.26762.r124822

**Published:** 2025-06-28

**Authors:** Yu-Feng Huang

**Affiliations:** 1 National Chung Hsing University, Taichung, Taiwan

The manuscript presents a chromosomally complete genome assembly of the Dark Umber moth, *Philereme transversata*, as part of the Darwin Tree of Life Project. The study outlines the collection, identification, sequencing, assembly, and annotation processes, employing both PacBio HiFi long-read sequencing and Illumina Hi-C short-read data. The resulting assembly spans 591.75 Mb, with 99.1% of the sequence scaffolded into 20 chromosomal pseudomolecules, including the Z sex chromosome. The mitochondrial genome was also assembled. Annotation performed by Ensembl identified 12,207 protein-coding genes and 1,934 non-coding genes. The manuscript provides a detailed account of laboratory methods, quality control, and data availability, offering a valuable resource for Lepidoptera genomics and biodiversity research.

 However, several issues should be addressed to improve the manuscript's clarity and scientific rigor:

**Typographical and Formatting Issues:**

- **Background Section**:
  - Paragraph 1: The sentence “It is present throughout Europe.” is overly brief and lacks context. Consider expanding to clarify ecological relevance or distribution.
  - Paragraph 3: Typo in “to .to support research...” — remove the duplicated "to".
- **Methods – Assembly Quality Assessment**:
  - Paragraph 2: The sentence “It aligns the PacBio reads in SAMtools (Danecek et al., 2021) and minimap2 (Li, 2018) and generates coverage tracks for regions of fixed size.” should be corrected to:
*“The PacBio reads were aligned using Minimap2 (Li, 2018), and SAMtools (Danecek et al., 2021) was used to compute coverage tracks for fixed-size genomic windows.”*

**Unit Consistency:**

- Units such as Mb and Gb should be used consistently and appropriately throughout the manuscript. In particular, the size of the mitochondrial genome should be reported in **base pairs (bp)**, not in megabases or gigabases, to reflect its smaller size accurately.

Addressing the issues above will enhance the manuscript's clarity, readability, and scientific value.

Are sufficient details of methods and materials provided to allow replication by others?

Partly

Is the rationale for creating the dataset(s) clearly described?

Yes

Are the datasets clearly presented in a useable and accessible format?

Yes

Are the protocols appropriate and is the work technically sound?

Partly

Reviewer Expertise:

bioinformatics, next-generation sequencing, genome assambly and gene annotation

I confirm that I have read this submission and believe that I have an appropriate level of expertise to confirm that it is of an acceptable scientific standard, however I have significant reservations, as outlined above.
